# Breath Hydrogen as a Biomarker for Glucose Malabsorption after Roux-en-Y Gastric Bypass Surgery

**DOI:** 10.1155/2015/102760

**Published:** 2015-10-11

**Authors:** Iman Andalib, Hiral Shah, Bikram S. Bal, Timothy R. Shope, Frederick C. Finelli, Timothy R. Koch

**Affiliations:** ^1^Department of Medicine, MedStar-Washington Hospital Center and Georgetown University School of Medicine, Washington, DC 20010, USA; ^2^EPGI, Allentown, PA 18104, USA; ^3^Center for Gastrointestinal and Liver Diseases, Centra Southside, Farmville, VA 23901, USA; ^4^Department of Surgery and Center for Advanced Laparoscopic & Bariatric Surgery, MedStar-Washington Hospital Center and Georgetown University School of Medicine, Washington, DC 20010, USA

## Abstract

*Objective*. Abdominal symptoms are common after bariatric surgery, and these individuals commonly have upper gut bacterial overgrowth, a known cause of malabsorption. Breath hydrogen determination after oral glucose is a safe and inexpensive test for malabsorption. This study is designed to investigate breath hydrogen levels after oral glucose in symptomatic individuals who had undergone Roux-en-Y gastric bypass surgery.* Methods*. This is a retrospective study of individuals (*n* = 63; 60 females; 3 males; mean age 49 years) who had gastric bypass surgery and then glucose breath testing to evaluate abdominal symptoms.* Results*. Among 63 postoperative individuals, 51 (81%) had a late rise (≥45 minutes) in breath hydrogen or methane, supporting glucose malabsorption; 46 (90%) of these 51 subjects also had an early rise (≤30 minutes) in breath hydrogen or methane supporting upper gut bacterial overgrowth. Glucose malabsorption was more frequent in subjects with upper gut bacterial overgrowth compared to subjects with no evidence for bacterial overgrowth (*P* < 0.001).* Conclusion*. These data support the presence of intestinal glucose malabsorption associated with upper gut bacterial overgrowth in individuals with abdominal symptoms after gastric bypass surgery. Breath hydrogen testing after oral glucose should be considered to evaluate potential malabsorption in symptomatic, postoperative individuals.

## 1. Introduction

The prevalence of overweight and obese individuals is increasing worldwide. A recent study demonstrated that, worldwide through 2013, the prevalence of adults with a body mass index of 25 kg/m^2^ or greater had risen to include 36.9% of men and 38.0% of women [1]. Second to tobacco, obesity is purported to be the most preventable cause of death in North America [2, 3]. Morbid obesity has been directly associated with significant comorbid conditions, and bariatric surgery remains the cornerstone of effective long-term treatment [4, 5].

Roux-en-Y gastric bypass (RYGB) has been the gold standard bariatric procedure, with 60–70% excess weight lost at two years, as well as significant improvement in glycemic control in diabetic patients [6]. However, most patients experience abdominal symptoms including abdominal pain, bloating, flatulence, and belching after bariatric surgery [7].

In patients after RYBG, quantitative bacteriology has revealed elevated populations of bacteria in both the gastric pouch and the bypassed stomach [8–10]. Using the “gold standard” of quantitative bacteriology, bacterial overgrowth, a known cause of malabsorption, has been identified even in patients with no abdominal symptoms after RYGB [9, 10]. This finding supports the notion that expensive quantitative bacteriology is not a good screening test to look for evidence of upper gut bacterial overgrowth in patients who have undergone RYGB.

After an overnight fast, measurements of breath hydrogen and methane before and after intake of oral glucose are both a safe and inexpensive test compared to quantitative bacteriology. The glucose-hydrogen breath test has a reported sensitivity of 44% to 90% and specificity of 78% to 83% compared to quantitative bacteriology for identifying colonic-type bacteria performed on aspirates from the small intestine [11–16]. Individual studies are difficult to compare due to differences in the quantitative culture supporting upper gut bacterial overgrowth (either > or = 10^5^ colony forming units or CFU/mL or > or = 10^6^ CFU/mL), differences in doses of glucose used (up to 75 grams), and differences in the rise in breath hydrogen accepted as supporting upper gut bacterial overgrowth (range is mostly a rise in breath hydrogen of 10 to 15 parts per million).

Several studies have validated the role of the glucose-hydrogen breath test in identification of small intestinal glucose malabsorption. The pathophysiology of the neonatal diarrheal illness glucose-galactose malabsorption has been shown to involve a defect in the intestinal brush border Na+/glucose cotransporter (SGLT1) [17]. In glucose-galactose malabsorption syndrome [18], abnormal breath hydrogen levels after glucose intake have been compared to abnormal 14C-glucose transport studies in jejunal mucosal biopsy specimen. These findings support the role of the glucose-hydrogen breath test in confirmation of small intestinal glucose malabsorption. In a second study involving patients following partial gastrectomy [19], pulmonary hydrogen measurements revealed that ten postgastrectomy patients with diarrhea failed to absorb all of an oral dose of glucose, while forty healthy controls and ten postgastrectomy patients with no diarrhea fully absorbed oral doses of 100 grams of glucose. The breath hydrogen results in this study [19] were compared to results of constant perfusion studies involving the terminal ileum, and so this study supported the presence of a symptom (diarrhea) in patients after partial gastrectomy who had evidence for small intestinal malabsorption of glucose.

In studies of patients who have undergone RYGB, an early study from 1981 revealed abnormal breath hydrogen levels after glucose ingestion in six of eight patients (75%) after RYGB [20]. In this study, fermentation gases were measured in one-half of the RYGB patients after exposure of jejunal secretions (obtained from RYGB patients) to carbohydrate [20]. This particular bioassay validated the presence of upper gut bacterial overgrowth leading to production of fermentation gases in patients after RYGB. In a prior study of patients with low blood thiamine levels after RYGB, we reported that 21 out of 21 patients had a rise of at least 15 ppm breath hydrogen within 30 minutes after a 50-gram oral dose of glucose [21].

Based upon these prior studies, the hypothesis of this present study is that breath testing before and after intake of oral glucose is effective for the identification of evidence of glucose malabsorption in individuals with abdominal symptoms after RYGB.

## 2. Subjects and Methods

### 2.1. Subjects

**Figure 1:**
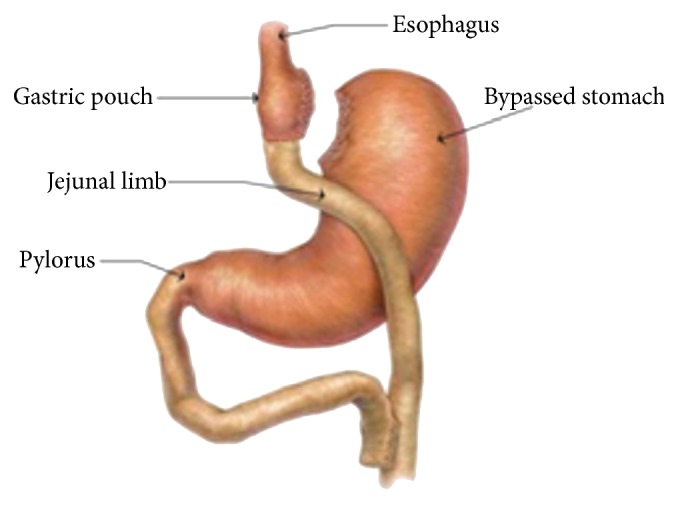
Roux-en-Y gastric bypass surgery. The orientation of the gastrojejunal anastomosis may be retrocolic or antecolic. In our experience, a retrocolic approach is more common after an open surgical procedure, while an antecolic approach is more common after a laparoscopic approach to surgery. The bypassed stomach and the loop of small intestine (termed the biliopancreatic limb) distal to the pylorus but proximal to the jejunojejunal anastomosis may function as a blind pouch.

This human study was approved by the Human Studies Subcommittee of MedStar Research Institute (Hyattsville, MD) on December 10, 2013. This is a retrospective study of patients who underwent Roux-en-Y gastric bypass surgery (see Figure 1). As shown in Figure 1, the bypassed stomach and the loop of small intestine (termed the biliopancreatic limb) that is distal to the pylorus but proximal to the jejunojejunal anastomosis may function as a blind pouch. Glucose breath testing was performed from 2006 to 2014 at MedStar-Washington Hospital Center, a large, urban community teaching hospital. Patients' medical charts were reviewed and patients who met the inclusion criteria were further assessed. We included sixty-three individuals who underwent hydrogen breath test with intake of oral glucose for evaluation of postoperative symptoms, which included abdominal pain, nausea, vomiting, early satiety, diarrhea, abdominal distension, and dyspepsia after RYGB. We also recorded the patient's demographics, the percentage of excess body weight (determined by patient weight − ideal body weight) lost (determined by weight lost/excess body weight) at 3 years after bariatric surgery, the results of glucose breath testing, and the time period between bariatric surgery and breath testing.

### 2.2. Glucose Breath Testing

We have previously described performance of glucose-hydrogen/methane breath testing in individuals with thiamine deficiency after RYGB [21]. No antibiotic use was permitted for 1 month before the breath test. Patients presented for breath testing after an overnight 12-hour fast prior to the procedure. Using a breath microlyzer (Quintron Instrument Company, Milwaukee, WI), baseline end expiratory values of breath hydrogen and methane were obtained. Each patient then received a solution containing 25 grams of glucose dissolved in 240 mL of water, which was ingested orally. The dose of glucose has been limited in our clinical laboratory to 25 grams as the result of RYGB patients noting marked abdominal pain and/or diarrhea after receiving a 50-gram dose of glucose. Breath hydrogen concentrations and methane concentrations were measured every 20 minutes for 2 hours. Based upon a previous study [15] that described at least a 10-part-per-million rise in breath hydrogen after 75 grams of glucose in individuals with > or = 10^6^ CFU/mL in jejunal aspirates, the results of our breath test were considered to be consistent with upper gut bacterial overgrowth, if the hydrogen or methane concentration increased by 10 or more parts per million in ≤30 minutes [21]. This early rise in breath hydrogen or methane was accepted as reflecting glucose fermentation by the bacteria in the upper gastrointestinal tract. A late rise of 10 or more parts per million above the plateau breath level in breath hydrogen or methane concentrations measured at ≥45 minutes was accepted as supporting glucose fermentation by either terminal ileal or colonic bacteria. If there was an early rise in breath hydrogen, a later rise in breath methane was not accepted as consistent with glucose malabsorption due to the potential for production of methane from hydrogen by intraluminal bacteria.

### 2.3. Statistics

Statistical analysis was performed using a standardized computer program (StatView; SAS Institute Inc., Cary, NC). The potential association between glucose malabsorption (late rise) and SIBO (early rise) was evaluated using Fisher's exact test. A *P* value of less than 0.05 was considered to be statistically significant.

## 3. Results

### 3.1. Clinical Characteristics

The average age of these subjects was 49 (range: 28 to 71) years old. This was a female predominant (95%) study. The average (±SEM) body mass index at the time of glucose breath testing was 35 ± 10 kg/m^2^ with a range of 21 to 54 kg/m^2^. The majority of subjects were Black Americans (70%), followed by Caucasian Americans (30%). Of the 63 individuals who had undergone RYGB, 21 subjects (33%) had been previously diagnosed with diabetes mellitus. The time period between RYGB and glucose-hydrogen breath testing averaged (±SEM) 65 ± 5 months. The baseline demographics for these study subjects are summarized in Table 1.

### 3.2. Breath Hydrogen Testing

**Figure 2:**
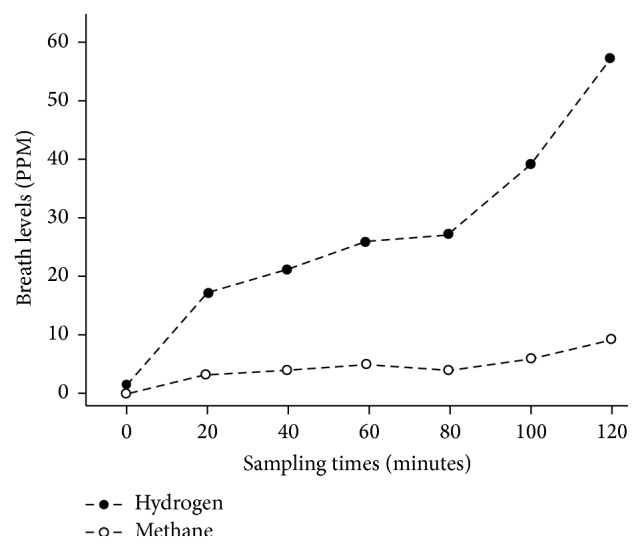
Representative subject in whom breath levels of hydrogen (closed circles) and methane (open circles) were measured; 25 grams of oral glucose was given after collection of breath at time 0. Note the initial rise in breath hydrogen (early rise), with a second rise in breath hydrogen (late rise) beginning at 80 minutes.

After receiving oral glucose, subjects could demonstrate a rise in breath hydrogen or methane of at least 10 parts per million at ≤30 minutes (consistent with upper gut bacterial overgrowth) or at least 10 parts per million above the plateau breath level measured prior to collection of breath at ≥45 minutes, as shown in the representative breath test in Figure 2. The presence of a late rise at ≥45 minutes supports unabsorbed glucose reaching the terminal ileum or colon, which would be consistent with glucose malabsorption. As shown in Table 2, out of the sixty-three subjects who had undergone RYGB, 51 (81%) had a rise in hydrogen or methane concentration of at least 10 parts per million at ≥45 minutes after glucose ingestion. Among these 51 individuals with a late rise supporting glucose malabsorption, 46 individuals (90%) also had an early rise in breath hydrogen or methane concentrations of at least 10 parts per million within 30 minutes after ingestion of glucose, supporting upper gut bacterial overgrowth. There was a significantly higher prevalence of glucose malabsorption in subjects with upper gut bacterial overgrowth when compared to subjects who did not have upper gut bacterial overgrowth (*P* < 0.001).

### 3.3. Weight Loss

As an apparent result of the process of conversion of the patients' medical records from paper charts to an electronic medical record, among the 63 individuals who had undergone RYGB, preoperative and 3-year postoperative weights were available in only 33 of these individuals. In the 33 individuals, 26 subjects were noted on glucose breath testing to have a late rise in breath hydrogen or methane (supporting the presence of glucose malabsorption), while 7 individuals had no late rise. In the 26 individuals with evidence for glucose malabsorption, the average percentage (±SEM) of excess body weight lost at 3 years was 66% (±3.8). In the 7 subjects with no findings supporting glucose malabsorption, the average percentage (±SEM) of excess body weight lost at 3 years was 56% (±8.1). With this limited information, there was no increase in the percentage of excess weight loss in subjects with evidence for glucose malabsorption (unpaired *t*-test: *P* = 0.24).

## 4. Discussion

Among patients with abdominal symptoms after RYGB, 81% had evidence for malabsorption by breath testing performed before and after receiving oral glucose. These findings support our hypothesis that glucose-hydrogen breath testing can function as a safe and inexpensive test for the examination of patients with abdominal symptoms after RYGB. Further clinical studies are underway to determine whether an abnormal hydrogen breath test after RYGB improves treatment outcomes in individuals with postoperative abdominal symptoms.

In our original study of upper gut bacterial overgrowth in individuals with thiamine deficiency after RYGB, we used a 50-gram dose of glucose [21], a dose which has been validated by other investigators [11, 12] for the diagnosis of upper gut bacterial overgrowth. Due to complaints among people who had undergone RYGB of uncomfortable abdominal symptoms and/or diarrhea after a 50-gram dose of glucose, we reduced our standard dose of glucose to 25 grams by mouth. A lower dose of glucose would not be expected to lower the specificity of the results of breath hydrogen and methane testing, but a lower dose of glucose could reduce the sensitivity of this test.

Studies have however shown that, in individuals with a rapid oral-cecal transit of <75 minutes, the glucose breath test may not be of benefit due to false positive results [22]. There are multiple lines of evidence against the presence of rapid oral-cecal transit in individuals who have undergone RYGB. First, oral-cecal transit has been measured after RYGB by Dirksen et al. using a scintigraphic technique and these authors reported fast gastric pouch emptying, but a delay in small intestinal transit time in individuals who had undergone RYGB [23]. These results are consistent with results reported by the Mayo Clinic (Rochester, MN) group [24]. In their study, individuals after RYGB (*n* = 19) could have accelerated gastric pouch emptying, but emptying of liquids from the Roux limb was slower than emptying of an anatomically equivalent segment of jejunum in controls [24]. The *T* ^1/2^ for colonic filling for RYGB patients ranged from 159 to 897 minutes but ranged from 16 to 370 minutes among ten healthy controls [24]. Second, surveys of individuals after RYGB show a low prevalence (4%) of dumping syndrome [25], a disorder that is supportive of rapid oral-cecal transit. A study using a recent dumping symptom scale also supported only a 12% prevalence of dumping symptoms up to 2 years after RYGB [26].

Among 63 individuals after RYGB, the frequent finding (81%) of a late rise (≥45 minutes) in breath hydrogen or methane supports the presence of glucose malabsorption. We however cannot determine the percentage of glucose malabsorption by the use of glucose breath testing.

Our present findings are supported by similar findings of decreased glucose absorption in the jejunal Roux limb in rats after RYGB [27] and in obese Zucker rats [28]. In small intestine, there is a sodium-dependent glucose cotransporter (SGLT1) at the apical membrane of the enterocyte [29, 30]. A second glucose transporter, GLUT2, has been identified at the apical or at the basolateral side of the enterocyte membrane, and GLUT2 has been thought to be involved with simple glucose diffusion [30, 31]. In rat jejunum, however, GLUT2 is the principal route for glucose absorption and GLUT2 mediates facilitated glucose diffusion [32].

Stearns et al. [27] have demonstrated in a rat model of RYGB that duodenal exclusion reduced intestinal glucose absorptive function by up to 68% in the Roux jejunal limb. This reduction in intestinal glucose absorption was present despite increased transcription of SGLT1 [27]; however Western blots revealed multiple SGLT1 proteins of different molecular weights [27], supporting suppression of intestinal glucose absorptive function by posttranscriptional changes in intestinal SGLT1. Indeed, inhibition of SGLT1 by using Phlorizin has been shown to reduce glucose transport [27].

It has been additionally shown that glucose absorption is lower in obese compared to lean Zucker rats [28]. Using enterocytes isolated from lean and obese Zucker rats, there were similar capacities for utilization of ^14^C-labelled glucose. However, reconstituted brush border membrane vesicles from obese Zucker rats showed a higher *V*_max_ for glucose transport without changes in *K*_*M*_ when compared to lean Zucker rats. Since the rate of glucose transport in the reconstituted system was higher in the obese animals (and thus there is a higher capacity for glucose transport in obese Zucker rats), the mechanisms involved in modulating intestinal transport of glucose in lean and obese Zucker rats remained unclear.

Results from studies of glucose transporters and glucose absorption in humans are unclear and suggest variability in the surgical anatomy during performance of RYGB. In a multicenter study from Germany and Switzerland, Ritze and associates reported that, in 20 obese individuals after RYGB, jejunal GLUT2 mRNA expression (relative to 18S) was increased compared to 14 lean individuals as controls [33]. However, the authors performed no Western blot analysis and so it was uncertain whether there was abnormal posttranslational expression and/or processing of GLUT2.

In a second human study from Australia, Nguyen et al. reported a potential increase in mRNA expression of SGLT1 and GLUT2 in jejunal mucosal biopsies after RYGB in 11 subjects [34]. Their results are however difficult to interpret because the authors did not normalize their mRNA levels to a “housekeeper gene.” The authors compared their results in jejunal mucosal biopsies to duodenal biopsies obtained from control patients, without providing any information about the lengths of the biliopancreatic limbs in individuals who had undergone RYGB. It is therefore not possible to determine the relative locations of the biopsies taken from the small intestine in their subjects after RYGB. The authors did not perform Western blot analysis to determine whether there is altered posttranslational expression and/or processing of SGLT1 and GLUT2.

The authors [34] in addition collected plasma measurements of glucose and 3-O-methyl-D-glucopyranose (3-OMG) in these research subjects. The location of absorption of 3-OMG in their studies however remains unclear. Their study was designed to collect blood at regular intervals over 240 minutes [34]. The authors then demonstrate an area-under-the-curve for 3-OMG at 0–270 min. The interpretation of these results is more complex because gastrointestinal physiologists have known since the 1960s that subtotal gastric resection with formation of a gastroenterostomy is associated with higher postprandial peak blood glucose levels in response to hypertonic glucose [35]. These findings occur in the presence of higher glucose concentrations measured in jejunal aspirates from subtotal gastrectomy patients [35]. These authors also noted that it was not clear why only a percentage of patients after partial gastrectomy develop symptoms after receiving hypertonic glucose [35]. Similarly, other investigators have more recently reported that creation of a gastric pouch with formation of a gastroenterostomy results in higher peak blood glucose levels in the early postprandial period after receiving oral glucose or a mixed meal [36–38].

In a prior study, Nguyen et al. had reported, in 10 individuals who had undergone RYGB, that the transit of glucose to the cecum was only 26 ± 10 minutes [39]. This rapid transit to the cecum reported by Nguyen et al. [39] could be related to altered vagal innervation of the small intestine as the result of technical aspects of performance of RYGB or the rapid transit could be explained by the presence of long biliopancreatic limbs (with subsequently shorted jejunal Roux limbs and common channels). The surgical technique for the performance of RYGB was however not discussed by these authors.

The finding in our present study of a significantly higher prevalence of glucose malabsorption in subjects with upper gut bacterial overgrowth when compared to subjects who did not have upper gut bacterial overgrowth supports bacterial overgrowth as a potential mechanism for the development of glucose malabsorption. This hypothesis is supported by a prior study of small intestinal biopsies which showed that mucosal sodium-dependent glucose transport was reduced in individuals with evidence for bacterial contamination of the small intestine [40]. Normally, the development of upper gut bacterial overgrowth is prevented by gastrointestinal defense mechanisms including the intestinal immune system, the production of gastric acid, pancreatic enzymes, intraluminal bile, small intestinal motility, and the barrier function of ileocecal valve [13]. Failure of any of these defense mechanisms has been suggested to be an origin for development of bacterial overgrowth [13]. Diabetes mellitus with autonomic neuropathy is also a known cause of bacterial overgrowth [13]. In our present study 33% of individuals who underwent RYGB had been diagnosed with diabetes mellitus. It is therefore unlikely that the presence of diabetes mellitus explains the high percentage of individuals with bacterial overgrowth and suggests that the relative achlorhydria of the gastric pouch after RYGB may be a more important pathogenetic factor.

Because only a small percentage of individuals in our present study had normal glucose-hydrogen breath testing after RYGB surgery, it is not yet clear whether glucose malabsorption is an origin for calorie malabsorption and thus continued weight loss in morbidly obese individuals after RYGB. This question will require a much larger patient population to determine whether glucose malabsorption improves postoperative weight loss in individuals after bariatric surgery.

There are specific limitations to our present study. This is a retrospective study and so the results may not be generalizable to all patients who have undergone RYGB. Our RYGB patients do not routinely undergo a repeat glucose breath test after antibiotic treatment of bacterial overgrowth. The individuals in this study who underwent glucose breath testing had previously undergone RYGB and were then seen in consultation because of abdominal symptoms. Individuals with no abdominal symptoms following RYGB are not included in this present study. In addition, it is uncertain whether the finding of glucose malabsorption is the result of RYGB or the result of obesity. By performing preoperative and postoperative glucose-hydrogen breath testing on the same morbidly obese subjects and by evaluating the clinical response to oral antibiotic therapy, many of these remaining questions should then be better addressed.

In conclusion, patients with abdominal symptoms after RYGB demonstrate a high prevalence of glucose malabsorption when evaluated by hydrogen breath testing after receiving oral glucose. These findings support glucose-hydrogen breath testing as a safe and inexpensive test for the examination of patients with abdominal symptoms after RYGB. Further clinical studies are underway to determine whether an abnormal hydrogen breath test after RYGB improves treatment outcomes in individuals with postoperative abdominal symptoms.

## Figures and Tables

**Table 1:** Demographics of individuals after Roux-en-Y gastric bypass surgery undergoing glucose-hydrogen breath testing (*n* = 63).

Age	
Mean	49 years
Range	28–71 years
Gender	
Female	60 (95%)
Male	3 (5%)
Race	
Black Americans	44 (70%)
Caucasians	19 (30%)
Body mass index	
Mean ± SEM	35 ± 10 kg/m^2^
Range	21 to 54 kg/m^2^
Months between bariatric	
surgery and breath testing	
Mean ± SEM	65 ± 5 months
Range	6 to 228 months

**Table 2:** Number of individuals after Roux-en-Y gastric bypass surgery with an early rise of breath hydrogen or methane (SIBO^*∗*^) or a late rise of breath hydrogen or methane (glucose malabsorption)^*∗∗*^.

		Early rise in breath (≤30 minutes)	
		No	Yes
Late rise in breath	No	12	0
(≥45 minutes)	Yes	5	46

^*∗*^SIBO: Small Intestinal Bacterial Overgrowth.

^*∗∗*^The potential association between glucose malabsorption (late rise) and SIBO (early rise) was evaluated using Fisher's exact test: *P* < 0.001.
